# Phycocyanin from *Arthrospira platensis* as Potential Anti-Cancer Drug: Review of In Vitro and In Vivo Studies

**DOI:** 10.3390/life11020091

**Published:** 2021-01-27

**Authors:** Steffen Braune, Anne Krüger-Genge, Sarah Kammerer, Friedrich Jung, Jan-Heiner Küpper

**Affiliations:** 1 Institute of Biotechnology, Molecular Cell Biology, Brandenburg University of Technology Cottbus-Senftenberg, 01968 Senftenberg, Germany; steffen.braune@b-tu.de (S.B.); sarah.kammerer@b-tu.de (S.K.); friedrich.jung@b-tu.de (F.J.); 2 Department of Healthcare, Biomaterials and Cosmeceuticals, Fraunhofer-Institute for Applied Polymer Research (IAP), 14476 Potsdam-Golm, Germany; anne.krueger-genge@iap.fraunhofer.de; 3 Carbon Biotech Social Enterprise AG, 01968 Senftenberg, Germany

**Keywords:** phycocyanin, *Arthrospira platensis*, cancer, tumor, drug, in vitro, in vivo

## Abstract

The application of cytostatic drugs or natural substances to inhibit cancer growth and progression is an important and evolving subject of cancer research. There has been a surge of interest in marine bioresources, particularly algae, as well as cyanobacteria and their bioactive ingredients. Dried biomass products of *Arthrospira* and *Chlorella* have been categorized as “generally recognized as safe” (GRAS) by the US Food and Drug Administration (FDA). Of particular importance is an ingredient of *Arthrospira*: phycocyanin, a blue-red fluorescent, water-soluble and non-toxic biliprotein pigment. It is reported to be the main active ingredient of *Arthrospira* and was shown to have therapeutic properties, including anti-oxidant, anti-inflammatory, immune-modulatory and anti-cancer activities. In the present review, *in vitro* and *in vivo* data on the effects of phycocyanin on various tumor cells and on cells from healthy tissues are summarized. The existing knowledge of underlying molecular mechanisms, and strategies to improve the efficiency of potential phycocyanin-based anti-cancer therapies are discussed.

## 1. Introduction

**Figure 1:**
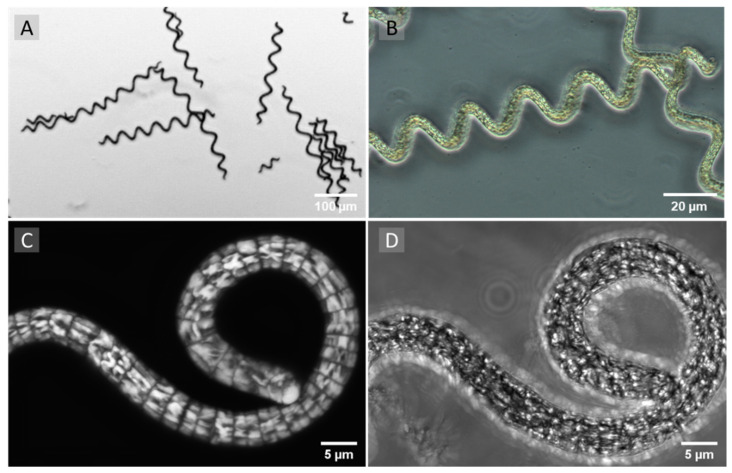
(**A**,**B**) Representative phase contrast images of unfixed *Arthrospira platensis* (*AP*). (**C**) Orthogonal projection of a three-dimensional laser scanning microscopy image stack (47 single images) of *AP*. Label-free laser scanning microscopy of an unfixed sample. The sample was exited at a 555 nm wavelength. Emissions were detected between 650 nm and 700 nm. (**D**) Transmitted mode image of the same position. Images were taken at 100-fold primary magnification with an Axio Observer.Z1/7 (Carl Zeiss Microscopy, Jena, Germany).

Over the last few decades, products from natural, non-synthetic origin have become increasingly important for the prevention and treatment of cancer due to the toxic side effects of many synthetic anti-cancer drugs [1,2,3,4]. *Arthrospira platensis* (AP), often called “Spirulina”, belongs to the phylum of cyanobacteria with characteristic photosynthetic capability [5]). These filamentous, gram-negative cyanobacteria or blue-green algae are considered as one of the sources of such natural bioactive substances (Figure 1) [6,7].

Up to now, *in vivo* toxicology studies of AP have not revealed any toxic effects on kidney, liver, reproductive system, or body physiology during or after the administration of acute or chronic doses [8,9,10]. A safety evaluation by the United States Pharmacopoeia—based on a 1966 to 2009 PUBMED literature review—and adverse event reports of the United States Food and Drug Administration (FDA) concluded that AP has a Class A safety [11].

Several dried biomass products of AP have also been categorized as “generally recognized as safe” (GRAS) by the FDA. A recommended dosage for adults is usually in the range of 3–10 g of AP per day. Maximally applied AP doses of 30 g/day did not lead to any negative side effects [12]. The regular consumption of considerably lower AP dry mass (but also phycocyanin) was shown to reduce intestinal inflammation, to improve the permeability of the intestinal tissue, and to increase the diversity of the intestinal microbiota e.g., in high-fat-diet rats (e.g., 3 g of AP per day) but also in apparently healthy mice (e.g., 2.1 g of AP per day) [13,14,15,16].

Analyses of the amino acid composition showed that AP is nutritionally at least comparable to soy, and close to the World Health Organization/Food and Agriculture Organization of the United Nations’ (WHO/FAO) standard of optimal protein intake [17]. In addition, AP is considered to be a source of minerals, vitamins and anti-oxidants including phycocyanin (PC), carotenoids, tocopherols and phenolic compounds [6,7,12,18,19,20,21,22,23,24,25]. Depending on the production and extraction process, two of the ingredients are described to affect tumor cells: PC and exopolysaccharides. However, since Challouf et al. were recently able to show that extracellular polysaccharides are not present in aqueous extracts and had no cytotoxic effect on tumor cells [26], PC can be considered a key active substance. Further ingredients that affect cell functions are chlorophyll, phycoerythrin, vitamin C, γ-linoleic acids, and α-tocopherol [27,28,29,30,31]. The latter are only present in minute quantities in AP or are not described to affect tumor cells.

PC is an oligomeric protein consisting of equal numbers of α and β subunits (with a molecular weight of about 18 and 21 kDa, respectively) [32,33]. The αβ-pairs mostly build the pigment as a trimer (αβ)3 or hexamer (αβ)6. Both α and β subunits have a bilin chromophore, which contains linear tetrapyrrole rings that are attached to the cysteine amino acid of the apoprotein by thioether linkages [34]. Medical applications of PC are of interest due to its anti-inflammatory, anti-viral, anti-cancer, immunostimulatory and anti-oxidant properties [35].

Recent anti-cancer studies of PC revealed a significant inhibitory effect on the growth of cancer cells in a time- and dose-dependent manner. Multiple mechanisms have been found, including the induction of apoptosis, cell cycle arrest, inhibition of DNA replication and the generation of reactive oxygen species (ROS) [32,36,37,38]. While apoptosis was significantly increased in cancerous cells, PC had a considerably lower toxicity on cells from healthy tissues, which makes it an appropriate candidate for chemotherapeutic applications [35,39,40].

In the present review, we summarize the effects of PC on cells that originate from various tumors, or on cells from healthy tissue in *in vitro* and *in vivo* studies. In addition, the existing knowledge of underlying molecular mechanisms are discussed.

## 2. Anti-Cancer Effects of Phycocyanin

PC is a blue-red fluorescent (~650 nm emission), water-soluble, non-toxic biliprotein pigment [33,41]. It is reported to be the main active ingredient of AP [42] and has been shown to have therapeutic properties, including anti-cancer activities [43,44,45]. At the cellular level, basic characteristics of tumor cells include unregulated cell proliferation, cellular immortalization, invasive cell growth, and in many cases, loss of capability for apoptosis [46]. The pharmacological effects of cytostatic medications in general aim to inhibit tumor cell proliferation by cell cycle arrest or induction of tumor cell death. Most cytostatic drugs are derived from natural compounds [47].

Accumulating evidence suggests that PC has a potent anti-cancer effect on various cancer types (such as breast cancer [48,49], liver cancer [50], lung cancer [51,52], colon cancer [53], leukemia [42] and bone marrow cancer [54]) *in vitro* and *in vivo*. On the other hand, even high-dose PC treatment does not induce significant toxic symptoms or mortality in animal experiments [55,56].

Table 1 summarizes results of *in vitro* studies concerning analyzed tumor types, used cell lines, the PC concentrations for the cell growth experiments and application times, cell proliferation, half maximal inhibitory concentrations (IC50) and cell morphology. Although concentrations and exposure times—and possibly also PC purity—differed greatly between the studies, the results clearly showed cell cycle arrest, and often, apoptosis/necrosis of the various tumor cells was induced [55]. In contrast, PC had almost no or even slight proliferative effects on cells originating from normal tissue [35].

## 3. Molecular Mechanisms of Phycocyanin-Induced Cell Death in Tumor Cells

**Figure 2:**
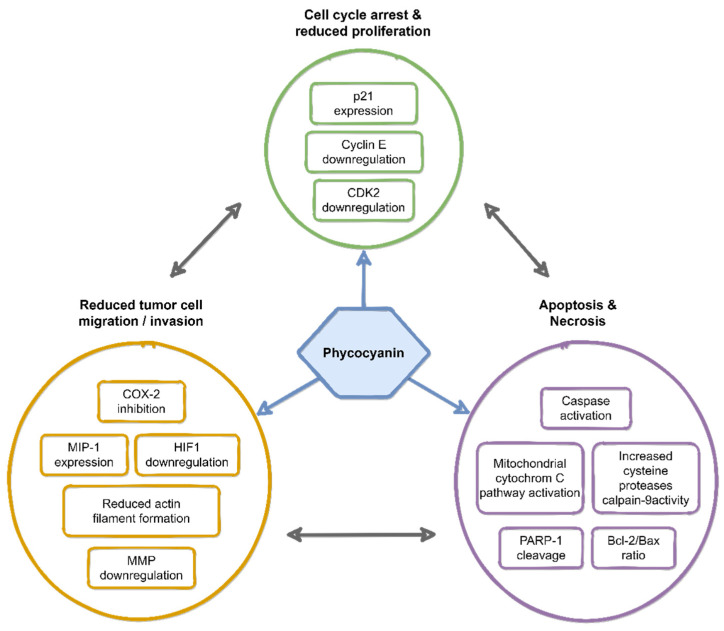
Overview of the reported molecular mechanisms of phycocyanin-induced anti-cancer activity.

**Figure 3:**
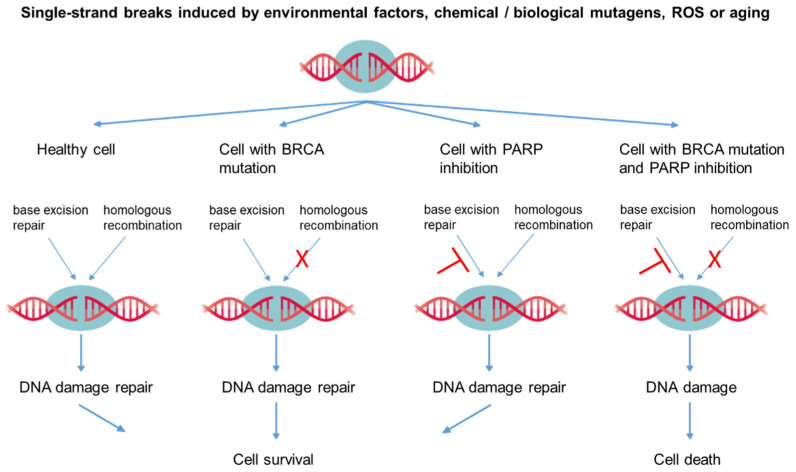
The role of gene defects (e.g., BRCA) and poly (ADP-ribose) polymerase (PARP) inhibition in the process of apoptosis. As an example, the repair of two independent single-strand breaks (or clustered single-strand breaks) is demonstrated (T = inhibited, X = blocked). ROS: reactive oxygen species.

An important mechanism to treat tumors is the induction of cell apoptosis. Various pathways have been described by which PC can impair tumor cells and induce apoptosis [57]. Mechanistically, PC exerts its anti-cancer activity by reducing cell proliferation and migration and inducing apoptosis, as well as cell cycle arrest (Figure 2). Obviously, PC can penetrate the cell membrane of *in vitro* cultivated tumor cells since it was found in the cytosol of HepG2 cells after treatment [68]. Localized near mitochondria, it was shown to induce apoptosis and necrosis via mitochondria-dependent intrinsic pathways. Li et al. demonstrated that PC inhibited the growth of HeLa cells in a dose-dependent manner [58]. It activated the mitochondrial cytochrome c pathway by altering the Bcl-2/Bax ratio (Bcl-2, anti-apoptotic protein; Bax, pro-apoptotic protein; Bcl-2/Bax ratio represents the degree of apoptosis induction). Further, activation of caspases and induction of poly (ADP-ribose) polymerase-1 (PARP-1) cleavage was shown by Subhashini and coworkers [42]. PARP-1 cleavage and inactivation might disable the immediate cellular response to DNA damages such as DNA excision repair [69]. After the supplementation of PC to the supernatant of tumor cells, remarkable morphological changes were observed. These comprised cell shrinkage, formation of membrane blebs, nuclear and cytoplasmic condensation, endolytic cleavage of the DNA into small oligo-nucleosomal fragments, formation of apoptotic bodies, and micronuclei characteristic of apoptosis (Figure 3). These results are well in line with earlier studies in which cancer cells treated with anti-cancer agents showed typical morphological signs of apoptosis, such as shrinkage and membrane bleb formation [58,64,70].

PC also affected tumor cell invasion. In MDA-MB-231 breast cancer cells, actin filaments were reduced while the migration potential decreased with PC supplementation [65]. PC is an inhibitor of cyclooxygenase 2 (COX-2), which converts arachidonic acid to prostaglandins and plays a key role in tumor progression and chemical resistance [71,72,73]. Prostaglandin-E2 is a tightly regulated product of COX-2, which promotes angiogenesis [74,75]. COX-2 inhibitors also up-regulated E-cadherin expression in Caco2 colon cancer cells [76]. Moreover, COX-2 was positively correlated with tumor invasion, metastasis, and poor prognosis in non-small cell lung cancer.

Matrix metalloproteases (MMP-2 and MMP-9), which are required for the invasion into surrounding tissues and tumor metastasis, were shown to be down-regulated by PC [77,78]. Furthermore, down-regulation of HIF-1 was shown, which is associated with increased oxygen demand and angiogenesis as well as MCP-1 expression (which is positively correlated with metastatic prognosis in the tumor environment). This down-regulation also promoted MIP-1 expression (which plays a role in reducing angiogenesis) [77].

Similarly, PC induced an increase in calpain-9 activity in colon cancer cells, a cysteine protease, which increases intracellular Ca^2+^ concentration [78]. It thus contributes to drug-mediated apoptosis by down-regulating the peroxisome proliferator-activated receptor γ (PPARγ) expression, which is related to tumor progression. Further anti-tumor mechanisms included inhibition of the colon cancer Wnt/β-catenin signaling and down-regulation of peroxisome proliferator-activated receptors α and δ expression (PPARα, PPARδ) [79].

Cell cycle regulation is of importance in normal cell proliferation, differentiation, and apoptosis, whereby dysfunction of cell cycle regulation is closely related to tumor development [46]. While the normal cell cycle is well regulated, tumor cells might proliferate infinitely. The cell cycle includes three major checkpoints which must be overcome successfully for cell division: the G1/S checkpoint, the G2/M checkpoint, and the spindle checkpoint. Failure to pass one or more of these checkpoints leads to cell cycle arrest and eventually to apoptosis. Supplementation of the cell culture medium with PC led to an arrest in the G0/G1 phase for colorectal tumor cells HT-29 and lung cancer cells A549. The DNA synthesis was thus blocked, and tumor cell proliferation inhibited [63]. Different groups reported that PC could also block G2/M cell cycle progression. This was found for pancreatic cancer cells PANC-1 [41], for human ovarian cancer cells SKOV-3 [79,80] and hepatoblastoma cells HepG2 [68].

Human breast cancer cells MDA-MB-231 were found to have different degrees of cell cycle arrest in the G0/G1 phase [29,47] by expression of cyclin-dependent kinase (CDK) inhibitor 1 (p21) as well as by down-regulation of Cyclin E and CDK2 expression [65]. Moreover, PC was shown to prevent leukemic cells (K562) from entering the S phase, and the cells were arrested in the G1 phase [35]. Altogether, these mechanisms induced by PC have the effect of inhibiting cell proliferation, and in turn, promoting apoptosis/necrosis [77].

The wide range of proliferation inhibition by PC from 3.7% to 100% in different cancer cell lines is striking. One hypothesis that could possibly explain this is the well-known genetic heterogeneity of different types of cancers, but there may also be substantial heterogeneity of cancer cells within a tumor. Forty years ago, Nowell reported that intra-tumor evolution via mutation and selection continues after tumor initiation [81]. Deficiencies in DNA repair are known to lead to higher mutation rates than in normal cells [82]. As a result, a tumor may contain a genetically diverse collection of subclones until it is clinically detectable [83]. Therefore, the intra-tumor heterogeneity can lead to the fact that tumor therapy can only lead a part of the tumor cells, but not all cells, into apoptosis/necrosis. This might be one reason why high intra-tumoral heterogeneity was associated with a poorer outcome in conventional tumor therapy in clinical studies [84,85,86].

## 4. Strategies for Potential Phycocyanin-Based Anti-Cancer Therapies

The effects of PC on tumor cell lines are nowadays quite substantially analyzed in multiple *in vitro* studies (see Table 1). The data of the sparse *in vivo* studies are summarized in Table 2. The dataset comprises studies with single and combinatory approaches (drugs and techniques), in oral as well as local treatments (Table 2). PC is currently not clinically used as an anti-cancer drug since the effects of a monotherapy seems to be not efficient enough [51]. In addition, the short *in vivo* half lifetime of PC puts some limitations to the application in medicine [87].

Very recently, Jiang and coworkers published a study to overcome these hurdles [57] by using a nanoparticle-based delivery system consisting of carboxymethyl chitosan—a material which has been shown to be water soluble, biodegradable, biocompatible and non-toxic [88]. In addition, they added a CD59 specific ligand peptide (CD59sp) to the nanoparticle for tumor targeting. CD59 was reported to be highly expressed in many solid tumors such as colon cancer [89], lung cancer [90], pancreatic cancer [91], and ovarian cancer [82,92], while CD59 was only marginally expressed in normal cells [90]. A first study revealed that the anti-tumor effects of the CD59sp containing the PC delivery system was more effective than the substance alone or the PC delivery system without targeting peptide *in vitro* (HeLa cells) as well as *in vivo* (female BALB/c nude mice, [57]). Besides carboxymethyl chitosan nanoparticles, liposome carriers have also been used to incorporate algal ingredients, and were shown to enhance cellular uptake [93].

Another strategy is the functionalization of nanoparticles with PC in combination with other substances, such as hematoporphyrin monomethyl ether for the noninvasive photodynamic anti-cancer therapy (PDT) [94,95]. In liver and breast cancer mouse model studies, PC-coated nanoparticles were injected in the tumor area and excited by near-infrared (NIR) laser light. This strategy allows the application of PC as a ROS-inducing photosensitizer and further allows for photoacoustic/thermal visualization of the treatment progress due to its fluorescence properties. The locally induced ROS generation results in apoptosis and necrosis. In combination with a further—thermal—treatment, tumor growth was shown to be decreased in these studies without systemic toxic side effects. However, the oral or local application of PC (without nanoparticle-based delivery system) was also shown to reduce tumor weight and forming rate in PDT [48,96].

The combination of PC with other natural compounds from marine organisms, which specifically supplement the effect on tumor cells via different mechanisms, seems to open further avenues for the therapy of tumors.

**Table 2:** Overview of *in vivo* studies investigating the influence of phycocyanin on the growth of various tumors.

Tumor Type	Tumor Induction	Animal Model	Combination Drug/Technique	Phycocyanin Concentration	Treatment Duration	Ref.
Lung	Injection of A549 cells (right flank)	Rat (nude)	Betaine	370.0 mg/kg of body weight per day, food supplement	28 days	[51]
Lung	Injection of A549 cells (armpit area)	Mouse (nude)	All-trans retinoic acid	0.2 mL (320 mg/mL) per day, injected in tumor area	10 days	[52,97]
Colon	Injection of 1,2-dimethyl-hydrazine dihydro-chloride (subcutaneous)	Rat, Sprague- Dawley	Piroxicam	Up to 200 mg/kg body weight per day, food suppl.	42 days	[77,98,99]
Ehrlich ascites carcinoma (EAC)	Injection of EAC cells (peritoneum)	Mouse, Swiss albino	Cisplatin	0.5 g/kg body weight of AP, food suppl.	14 days pre and 14 days post inocul.	[100]
Esophag. Squamous cell carc.	ESCC EC9706	Mouse		Injected in tumor area		[101]
Cervix	Injection of SiHa/HeLa cells (axillary fossa/ armpit area)	Mouse, BALB/c	Nanoparticles functionalized: CD95sp and PC	Injected in tumor area/tail vein once every 2 days	20 days	[57,102,103]
Liver	Injection of H22 cells (armpit area)	Mouse, BALB/c	Photodynamic therapy (PDT)	0.02 mL (10 mg/mL) per day injected in tumor area	10 days	[96]
Breast	Injection of MCF-7 cells (spleen area)	Mouse, BALB/c	PDT	2 mL (320 mg/mL) per day	13 days	[48]
Breast	Injection of MCF-7 cells (right abdomen)	Mouse, BALB/c	PDT, Nanoparticle coated with hematoporphyrin mono-methyl ether and PC	100 µL nanoparticle solution (3 µg/g) injected in tumor, per day, once every 2 days	14 days	[95]
Breast	Injection of 4T1 cells (subcutaneous)	Mouse, BALB/c	PDT, Nanoparticles functionalized with PC	100 µL nanoparticle solution (cor. 150 µg/mL PC)	14 days	[94]

First studies showed that PC can potentially improve the efficacy of currently available anti-cancer drugs [57]. The combination of PC and topotecan to human prostate adenocarcinoma cells (LNCaP) increased the activity of caspase-9 and caspase-3, increased free radical oxygen (ROS) levels, induced apoptosis of tumor cells, and reduced side effects of topotecan in a rat tumor model [104]. The combination of Piroxicam (a traditional non-steroidal anti-inflammatory drug) with PC in 1,2 dimethylhyadrazine (DMH)-induced rat colon carcinogenesis, showed a more than 70% higher effect than single-use drugs. DNA fragmentation increased and cyclooxygenase 2 (COX-2) expression and prostaglandin E2 (PGE-2) levels were significantly reduced. In addition, the number and size of tumors were also reduced [98,99]. The combination of all-trans retinoic acid (ATRA) with PC could significantly reduce the dose and side effects of ATRA in A549 pulmonary tumor cells. The combination therapy significantly down-regulated anti-apoptotic protein Bcl-2, up-regulated the expression of pro-apoptotic Caspase-3 protein, inhibited cell-cycle-related CDK-4 and Cyclin D1 protein expression, inhibited complement regulatory protein CD59 expression, and induced apoptosis in HeLa cells [52,97]. When lung cancer A549 cells were treated with betaine, A549 cell viability decreased by 50%, and the combination of betaine and PC decreased the viability by an additional 10–20% [51]. The NF-κB expression was reduced, the amount of pro-apoptotic protein p38 MAPK increased and a G2/M cell cycle arrest was induced [51]. In a very recent animal study in 220 female Swiss albino mice, Hashem et al. found that the combination of AP (0.5 g/kg b.wt., po) in addition to Cisplatin (40 µg/mouse/ip), an extensively used chemotherapeutic drug with broad-spectrum activity, promoted the apoptotic and cytotoxic functions of Cisplatin on the combination group against Ehrlich ascites carcinoma after two weeks of application [100]. Furthermore, AP significantly alleviated the Cisplatin-induced hematotoxic, hepatotoxic, and nephrotoxic impacts in normal mice.

These studies show that the addition of PC to already well-established anti-cancer drugs could improve cancer therapy significantly. In addition, the dosage of the cytostatic might be reduced, what often leads to less severe toxic side effects.

Beyond the mitigation of cancer activity, Ji and colleagues reported the influence of PC on the epithelial-to-mesenchymal transition (EMT) [105]. EMT is a pivotal and intricate process that increases the metastatic potential of cervical cancer. The authors induced EMT by TGF-β1 in cervical cancer cells. PC inhibited EMT in Caski cells by down-regulating N-cadherin and up-regulating E-cadherin protein expression through the TGF-β/smads signaling pathway. Furthermore, C-phycocyanin (C-PC) could inhibit the expression of Twist, Snail and Zeb1 transcription factors related to EMT. In addition, C-PC could inhibit the migration and invasion of Caski cells induced by TGF-β1. They concluded that C-PC reversed TGF-β1-induced epithelial-to-mesenchymal transition in cervical cancer cells and down-regulated the TGF-β/smad signaling pathway [105].

In this respect, it is of considerable importance that PC did not negatively affect non-malignant cells, so that high-dose therapies are possible (see Table 3). *In vivo* studies showed no toxicity, no adverse effects, and no mortality during acute toxicity tests of PC in rats and mice [106,107]. This applied even to the oral feeding of 3000 mg [106] or 5000 mg [52,108] per kg body weight. Intraperitoneal administration of 70 mg [109] and even 200 mg PC per kg body weight [110] also revealed no adverse effect in rats.

## 5. Effect of Phycocyanin on Tumor Cells in Comparison to Non-Malignant Cells

Why PC is toxic to tumor cells but non-toxic to non-malignant cells is still unclear and the subject of much debate. Different molecular pathways can be hypothesized, which might provide access to this enigma, and may help to solve it in the future. Differences in the function of non-malignant cells compared to tumor cells offer an approach to answer this question.

The underlying characteristic of cancer cells is the development of genomic instability, which promotes the development and accumulation of cancer-relevant mutations, finally leading to malignant transformation [46]. One difference between cancer cells and non-malignant cells consists of genetic or epigenetic changes that can lead to uncontrolled tumor growth. In healthy organisms, genomic stability is controlled by various repair mechanisms. Mammalian cells comprise a variety of repair mechanisms that potentially detect and repair DNA damages, such as single-strand breaks, base adducts, or base oxidations etc., and thus maintain genomic integrity. Important repair systems include base and nucleotide excision repair (BER, NER) as well as double-strand break repair by homologous recombination (HR) or non-homologous end-joining (NHEJ) [8,112]. The poly (ADP-ribose) polymerase (PARP) enzymes PARP-1 and PARP-2 recognize DNA single- and double-strand breaks via their zinc finger binding domains, and contribute to DNA repair by enzymatic activation of the BER or other mechanisms [10,11,12,113]. By inhibiting PARP, single-strand breaks are no longer repaired and double-strand breaks occur during the next cell division in the nucleus, which then leads to apoptosis.

In non-malignant cells, DNA damages can be repaired by homologous recombination. This context is shown in Figure 3.

PARP inhibitors, when used specifically in patients with BRCA gene defects, offer the possibility of attacking tumor cells and leading them to apoptosis, while in healthy cells in the same organism, the repair of single-strand breaks should continue (see Figure 3).

Reddy reported that PC has PARP-inhibiting properties possibly mediated by the release of cytochrome c from mitochondria with the activation of caspase 3 following apoptosis [114]. This cleavage of PARP might then preclude the catalytic domains of PARP and presumably disable PARP from coordinating subsequent repair and maintenance of genome integrity. This has already been achieved in PARP cleavage by PC-mediated apoptosis of K562 cells (lymphoblasts of chronic myelogenous leukemia patients) [42]. This clearly demonstrates that PARP inhibition with PC in combination with a gene defect such as BRCA can lead to the apoptosis of tumor cells.

For many of the tumor types shown in Table 1, mutations of BRCA1 and/or BRCA2 tumor suppressor genes are described [115,116,117,118,119]. Women with such BRCA mutations have a risk of 50–80% to develop breast cancer by the age of 70 [120], and they have a further risk of 40–65% to develop ovarian cancer. Men with BRCA mutations have a 2–7 times elevated risk for prostate cancer. Both sexes face a 2–4 times elevated risk for colon or pancreatic cancer [115,116,117,118,119]. AP might be able to induce apoptosis in those tumor types.

Patients with mutations in other key genes within the DNA damage repair pathway may also respond to treatment with PARP inhibitors, and identification of these alterations could significantly increase the percentage of patients that may benefit from PC [121]. Worth mentioning is the RAD51C gene (like BRCA1 located on chromosome 17), which was identified in more detail in 2010 [122]. After BRCA1 and 2, it is the only high-risk gene found so far, and is therefore named BRCA3 [123]. Defects also lead to a significant increase in risk (approximately the same as BRCA1/2): according to current knowledge, about 60% to 80% of women with RAD51C mutation develop breast cancer, and 20% to 40% develop ovarian cancer. Further gene defects associated with the moderate elevation of a risk to develop breast or ovarian tumor were identified in a population-based family study (COGS study [124]).

## 6. Conclusions

The data indicate that PC may be considered a safe drug to reduce or inhibit tumor cell growth. The combination with other anti-cancer drugs and/or radiation therapy might allow the reduction of the effective dose of established anti-cancer drugs, which would minimize dose-related side effects and improve the therapeutic outcome. Also, encapsulation of PC might prolong the half-lifetime, and could thus improve the effectiveness.

However, one should have in mind, that all the studies shown were performed using cancer cell lines. Prior to clinical use, studies involving primary tumor cells and animals must be performed to prove whether comparable results appear. A study using cells out of a tumor as well as cells from the neighbored healthy tissue is now in preparation and will show whether this hypothesis can solve the enigma of why AP leads to apoptosis of tumor cells but does not harm primary non-malignant human cells.

## Figures and Tables

**Table 1:** Effects of phycocyanin on the growth of various tumor cell lines. (-: not given).

Tumor Type	Cell Line	Phycocyanin Concentration		Application Time (h)	Proliferation (%)	IC50		Morphology	Ref.
Cervical carcinoma	HeLa	80; 200	µg/mL	72; 24	−32; −20	-; 1104	µg/mL	epithelial	[57,58]
Human colorectal adenocarcinoma	HCT116	50	µg/mL	48	−73	18.8	µg/mL	epithelial	[59]
Pancreatic adenocarcinoma	Capan-1	100	µM	72	−80	6.2	µM	epithelial	[55]
Pancreatic adenocarcinoma Pancreatic adenocarcinoma	BxPC3 PA-TU-8902	100 0.3	µM g/L	72 24	−100 −82	15.1 -	µM -	epithelial epithelial	[55,60]
Human ductal pancreas carcinoma	PANC-1	100	µM	72	−70	12.2	µM	epithelial	[55]
Hepatoblastoma	HepG2	100	µM	72	−76	13	µM	epithelial	[55]
Hepatoblastoma	HepG2	7; 50	µg/mL	24; 48	−61; −75	1.75; 22.3	µg/mL	epithelial	[59,61]
Prostate carcinoma	DU145	100	µM	72	−70	18	µM	epithelial	[55]
Large cell lung cancer Lung adenocarcinoma	H460 A549	100 50	µM µg/mL	72 24	−95 −38	14 99.2	µM µg/mL	epithelial epithelial	[55,62]
Alveolar adenocarcinoma	A549	60	µg/mL	48	−64	-	-	epithelial	[63]
Nsc broncho carcinoma	H1299	4.8	µM	24	−11.3	-	-	epithelial	[33]
Nsc broncho carcinoma	H460	4.8	µM	24	−3.7	-	-	epithelial	[33]
Nsc broncho carcinoma	LTEP-A2	4.8	µM	4	−14.5	-	-	epithelial	[33]
Human colorectal adenocarcinoma	HT-29	50; 200	µg/mL	48; 72	−63; −100	-	-	epithelial	[63,64]
Triple negative breast cancer	MDA-MB-231	20	µM	6	−82	5.98	µM	epithelial	[65]
Triple negative breast cancer	MDA-MB-231	294	µg/mL	24	−30	294	µg/mL	epithelial	[57]
Ductal carcinoma	BT-474	20	µM	6	−80	8.45	µM	epithelial	[65]
Breast cancer	MCF-7	20; 100	µM	6; 72	−58; −65	15.4; 33	µM	epithelial	[4,65]
Breast cancer	MCF-7	5.66	µg/mL	48	-	5.66	µg/mL	epithelial	[66]
Breast cancer	MCF-7	40	µM	72	−53	-	-	epithelial	[49]
Mammary gland adenocarcinoma	SKBR 3	20	µM	6	−60	17.7	µM	epithelial	[65]
Squamous cell carcinoma, cervix	SiHa	376	µg/mL	24	−35	376	µg/mL	epithelial	[57]
Malignant melanoma	A375	40	µM	72	−54	-	-	epithelial	[49]
Chronic myelogenous leukemia Histiocytic tumor	K562 AK-5	100 60	µM µM	96 72	−49 −70	- 60	- µM	lymphoblast macrophage	[42,67]

**Table 3:** Effects of phycocyanin on growth of non-tumorigenic cell lines and primary cells.

Cell Origin	Cell Type	Phycocyanin Concentration		Appl. Time (h)	Prolif. (%)	IC50		Morphology	Ref.
Ovary (Chin. Hamster)	CHO	20	µM	6	≈	-	-	epithelial	[58]
Mammary gland	MCF-10A	25	µg/mL	24	↑	>20	-	epithelial	[65]
Skin	HSF	1000	µg/mL	24	≈	-	-	fibroblast	[62]
Skin	CCD-986sk	40	µM	72	+42	-	-	fibroblast	[35]
Skin	Hs68	100	µM	72	≈	-	-	fibroblast	[49]
Liver	LO2	100	µM	72	≈	-	-		[55]
Liver	QSG-7701	100	µM	72	≈	-	-	epithelial	[55]
Human heart ventricle	AC-16	100	µM	72	≈	-	-		[55]
Kidney cortex	HK-2	100	µM	72	≈	-	-	epithelial	[55]
Peripheral blood	NK-82	100	µM	72	≈	-	-	NK cells	[55]
Endothelium of umbilical vein	HUVEC	100	µM	72	≈	-	-	epithelial	[55]
Endothelium of umbilical vein	HUVEC	50	µg/mL	80	+31	-	-	epithelial	[111]
Connective tissue (Mouse)	L929	596	µg/mL	24	−25	596	µg/mL	fibroblast	[57]

-: not given, ↑: increase, ≈: approximately equal/unchanged.
